# A nomogram for predicting the risk of new vertebral compression fracture after percutaneous kyphoplasty

**DOI:** 10.1186/s40001-023-01235-y

**Published:** 2023-08-11

**Authors:** Aiqi Zhang, Yichen Lin, Mingxiang Kong, Jiahao Chen, Wei Gao, Jiajun Fan, Junjie Wang, Zhe Chen

**Affiliations:** 1 The Second Clinical Medical College of Zhejiang, Chinese Medical University, Hangzhou, Zhejiang China; 2 Department of Orthopedics, Zhejiang Provincial People’s Hospital, Hangzhou, Zhejiang China; 3 Department of Orthopedics, The Second Affiliated Hospital of Zhejiang Chinese Medical University, Hangzhou, Zhejiang China

**Keywords:** Osteoporosis vertebral compression fractures, New vertebral compression fractures, Percutaneous kyphoplasty, Nomogram

## Abstract

**Background:**

New vertebral compression fractures (NVCFs) are common adverse events in percutaneous kyphoplasty (PKP). The present study aimed to investigate the risk factors for NVCFs in patients after PKP and to construct a nomogram for the prediction of the risk of re-fracture.

**Methods:**

We retrospectively analyzed the medical records of patients after PKP surgery between January 2017 and December 2020. Patients were divided into an NVCF group (n = 225) and a control group (n = 94) based on the presence or absence of NVCFs, respectively, at follow-up within 2 years after surgery. Lasso regression was used to screen for risk factors for re-fracture. Based on the results, a Lasso-logistic regression model was developed, and its prediction performance was evaluated using receiver operating characteristic curves, calibration, and decision curve analysis. The model was visualized, and a nomogram was constructed.

**Results:**

A total of eight potential predictors were obtained from Lasso screening. Advanced age, low body mass index, low bone mineral density, lack of anti-osteoporosis treatment, low preoperative vertebral body height, vertebral body height recovery ≥ 2, cement leakage, and shape D (lack of simultaneous contact of bone cement with the upper and lower plates) were included in the logistic regression model.

**Conclusions:**

A nomogram for predicting postoperative NVCF in PKP was developed and validated. This model can be used for rational assessment of the magnitude of the risk of developing NVCFs after PKP, and can help orthopedic surgeons make clinical decisions aimed at reducing the occurrence of NVCFs.

## Background

Osteoporosis, the most common bone disease, is a systemic condition characterized by decreased bone mass which leads to deterioration of bone tissue microarchitecture and increased bone fragility. Approximately one-third of women experience osteoporosis-related fractures after the age of 50 compared to one-fifth of men [1]. Osteoporosis has an insidious onset and is potentially very harmful. Falls or injuries in older adults often result in osteoporotic fractures, with vertebral compression fractures (VCFs) being the most common [2]. Patients with VCFs experience severe chronic pain, kyphosis, decreased mobility, reduced pulmonary function [3], and increased mortality rates [4]. Since 1987, osteoporotic vertebral compression fractures (OVCFs) have been widely treated with percutaneous kyphoplasty (PKP), which is effective in relieving pain and restoring vertebral height with minimal trauma and rapid recovery time; therefore, PKP has become the treatment of choice for OVCFs [5, 6].

However, re-collapse of cemented vertebrae frequently occurs after percutaneous augmentation [7]. Many risk factors for re-fracture have been considered, and several relevant factors have been reviewed in the literature [7, 8]. Very few studies have developed visual models for risk factors of re-fracture after PKP, and none of these studies have included bone mineral density (BMD) in these models [9, 10]. Previous studies have suggested that BMD has major effects on OVCFs [11], with a low BMD predisposing patients to re-fracture after PKP [7]. A retrospective study of cases by Venmans et al*.* [12] found that the severity of osteoporosis was a risk factor for vertebral re-fracture after PKP surgery. Therefore, in the present study, we developed and validated a nomogram to predict the risk of NVCFs after PKP surgery.

## Methods

### Patients

A total of 466 patients with single-segment OVCFs treated with PKP at Zhejiang Provincial People’s Hospital between January 2017 and December 2020 were analyzed. The inclusion criteria were: 1. single-segment OVCFs caused by low-energy injuries (falls, bending) in older patients treated with PKP, 2. significant low back pain or restricted movement, and 3. indications on imaging examination (radiography or computed tomography showing vertebral fracture; magnetic resonance (MR) T2-weighted imaging showing significant edema of the fractured vertebrae). The exclusion criteria were: 1. OVCFs due to cancer, infection, or tuberculosis; 2. long-term glucocorticoid treatment; 3. inability to tolerate surgery; 4. spinal cord compression with significant neurological symptoms such as numbness or muscle weakness; 5. multiple segmental fractures of the spine; 6. endocrine disorders such as hyper- or hypothyroidism; and 7. loss to follow-up for other reasons or incomplete preoperative imaging data.

Based on the inclusion and exclusion criteria, 147 patients were excluded: 66 did not meet the indications for single-segment vertebral fractures, 45 lacked complete preoperative imaging data (imaging data from outside institutions), 22 had malignant cancer, one received glucocorticoid treatment, and 12 were lost to follow-up. A total of 319 patients were enrolled in the study, including 225 patients in the training cohort and 94 patients in the validation cohort.

### Observation indicators

**Fig. 1:**
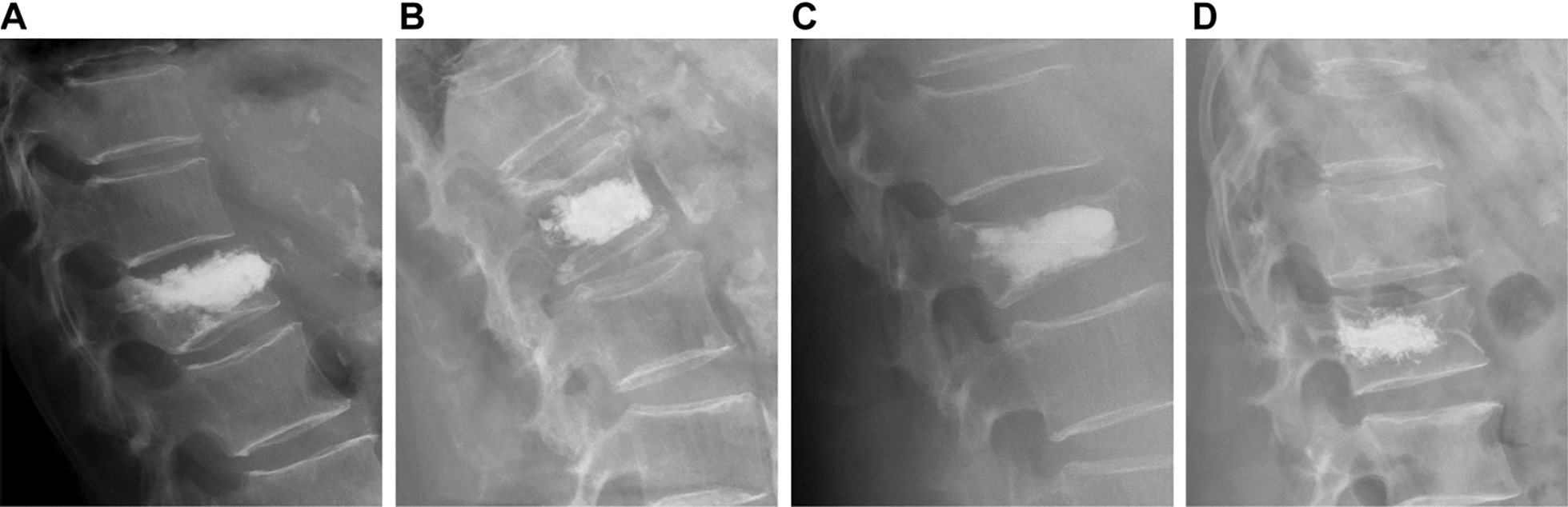
Distribution of the type of bone cement. **A** Shape A: Bone cement is only in contact with the upper endplate. **B** Shape B: Bone cement is only in contact with the lower endplate. **C** Shape C: Bone cement is in contact with the upper and lower endplate. **D** Shape D: Bone cement is not in contact with the upper and lower vertebral plates

We reviewed previously published literature on the risk factors that may contribute to VCF after PKP, as well as the general characteristics and imaging parameters of patients. Age, sex, history of diabetes and hypertension, body mass index (BMI), BMD, vertebral segments fractured, mean volume of cement used, volume of cement leakage, volume of cement dispersion, distribution of cement (Fig. 1), contact between cement and endplate, anti-osteoporosis treatment, scoliosis status, preoperative Cobb angle, postoperative Cobb angle, preoperative anterior vertebral height (AVH), and postoperative AVH of the patients were recorded. Good bone cement dispersion was defined as bone cement crossing the midline of the vertebral body on spinal X-ray; otherwise, it was defined as poor bone cement dispersion. Anterior vertebral body height ratio (AVHR) was defined as the percentile of the height of the anterior margin of the compressed vertebral body relative to the mean height of the anterior margins of the adjacent upper and lower vertebral bodies. The anterior vertebral height recovery ratio (AVHRR) was defined as postoperative AVHR minus preoperative AVHR. The Cobb angle was defined as the angle formed by the superior and inferior endplates of the fractured vertebral body. The Cobb angle recovery was defined as the percentile of the preoperative Cobb angle over the postoperative Cobb angle.

### Surgical methods

The patient was placed in the prone position, frontal C-arm fluoroscopy was used to locate the bilateral projections of the pedicles of the injured vertebrae, and marker lines were drawn. The patient was routinely disinfected and draped, and 1% lidocaine was used for local anesthesia at the bilateral pedicle puncture sites. The left side was operated on first, and an incision approximately 1 cm in length was made at the projection of the left pedicle. The skin and subcutaneous tissues were incised, and the pedicle was punctured at the projection at the 10 o'clock position approximately, paying attention to the sagittal angle and the internal angle. Frontal and lateral C-arm fluoroscopy was used to confirm satisfactory puncture. The puncture needle was withdrawn, a guide needle was introduced, a working trocar was placed approximately 0.5 cm anterior to the posterior margin of the vertebral body, and the guide needle was withdrawn. A reamer was placed in the working trocar, the reamer was withdrawn, and the bone was removed and preserved for pathological examination. A compression rod was placed, the rod was withdrawn, contrast agent was injected, and then a balloon was placed and expanded to an appropriate pressure. The contrast agent was withdrawn, and the balloon was removed. The same procedure was then performed by puncturing the right side of the projection of the pedicle at approximately 2 o’clock position. A mesh bag was then placed in each working trocar bilaterally, and bone cement was injected. These operations were performed under C-arm monitoring, and cement injection was immediately discontinued if intraoperative cement leakage was detected.

### Postoperative management

All patients received oral calcium supplements (600 mg/day) and active vitamin D (0.25 μg/dose, twice daily) as the foundation of treatment. Additionally, they were administered either intravenous infusion of zoledronic acid (5 mg/year) or subcutaneous injection of denosumab (60 mg/6 months). Patients wore a thoracolumbar brace for 4 months while out of bed and active. All patients underwent frontal and lateral spinal X-ray at 24 h after surgery. Follow-up was conducted at 1 month, 3 months, 6 months, 1 year, and 2 years after surgery. Telephonic follow-up was conducted for patients unable to visit for regular outpatient follow-up. Due to convenience and financial considerations, patients underwent frontal spine radiographs during follow-up. Spinal MR examination was used to support diagnosis of early NVCFs. All patients were followed up for 2 years with post-PKP re-fracture as the study endpoint.

### Evaluation of re-fractures

The principal diagnostic criteria for re-fracture after PKP were as follows: 1. development of new low back pain and/or restricted lumbar motion after PKP; 2. spinal MR examination suggesting new spinal fracture with low signal in T1 phase and high signal in T2 phase; and 3. X-rays and MR examination suggesting that the fractured vertebrae were different from the previously operated vertebrae.

### Statistical analysis

SPSS 26.0 and R (4.2.0) were used for statistical analysis of data. Continuous variables were expressed as mean ± standard deviation, and the independent samples t-test was used for comparison between groups. Categorical variables were expressed as percentages, and the chi-square test was used for comparison between groups. Lasso regression was used to screen for risk factors. Logistic regression was used to construct a prediction model based on the risk factors screened from Lasso regression. Risk factors were entered into a predictive nomogram prediction model using R software. Differences with p < 0.05 were considered statistically significant.

## Results

### Characteristics of patients

Patients were randomly divided into a training cohort and a validation cohort at a ratio of 7:3. The training cohort was used to construct the model and the validation cohort was used to test the model. In the training cohort, 32 of the 225 patients (14.2%) developed NVCFs, the control group had an average age of 74 ± 9 years and a T-score of -2.39 ± 1.23 kg/m^2^, and the NVCF group had an average age of 80 ± 7 years and a T-score of -3.00 ± 1.32 kg/m^2^. In the validation cohort, 11 of the 94 patients (11.7%) developed NVCFs, the control group had an average age of 74 ± 10 years and a T-score of 2.56 ± 1.37 kg/m^2^, and the NVCF group had an average age of 75 ± 10 years and a T-score of -3.55 ± 1.20 kg/m^2^. The clinical characteristics of the patients are shown in Table 1. No patients exhibited postoperative complications such as infection, nerve injury, vascular thrombosis, or bone cement reaction.

**Table 1:** Clinical characteristics of the training cohort and the validation cohort

Variables	Training cohort (n = 225)			Validation cohort (n = 94)		
	Control (n = 193)	NVCFs (n = 32)	p	Control (n = 83)	NVCFs (n = 11)	p
Sex, n (%)			0.819			0.205
Male	33 (17%)	6 (19%)		17 (20%)	0 (0%)	
Female	160 (83%)	26 (81%)		66 (80%)	11 (100%)	
Age (years)	74 ± 9	80 ± 7	< 0.001	74 ± 10	75 ± 10	0.826
BMI (kg/m^2^)	22.5 ± 3.7	20.9 ± 3.4	0.017	23.1 ± 3.8	21.5 ± 5.3	0.367
BMD	− 2.39 ± 1.23	− 3.00 ± 1.32	0.019	− 2.56 ± 1.37	− 3.55 ± 1.20	0.024
History of hypertension, n (%)			0.340			0.056
Yes	102 (53%)	14 (44%)		48 (58%)	3 (27%)	
No	91 (47%)	18 (56%)		35 (42%)	8 (73%)	
History of diabetes, n (%)			0.464			0.113
Yes	32 (17%)	7 (22%)		20 (24%)	0 (0%)	
No	160 (83%)	25(78%)		63 (76%)	100 (100%)	
Augmentation segment, n (%)			0.028			0.057
T5–7	4 (2.1%)	3 (9.4%)		6 (7.2%)	3 (27%)	
T8–12	43 (22%)	11 (34%)		23 (28%)	4 (36%)	
L1–5	146 (76%)	18 (56%)		54 (65%)	4 (36%)	
Bone cement dosage (mL)	4.94 ± 1.20	4.91 ± 0.90	0.834	4.64 ± 1.30	4.41 ± 1.30	0.582
Bone cement leakage, n (%)			0.016			0.463
Yes	44 (23%)	14 (44%)		20 (24%)	4 (36%)	
No	149 (77%)	18 (56%)		63 (76%)	7 (64%)	
Bone cement dispersion, n (%)			> 0.999			0.066
Yes	189 (98%)	32 (100%)		81 (98%)	9 (82%)	
No	4 (2%)	0 (0%)		2 (2%)	2 (18%)	
Bone cement distribution, n (%)			0.079			0.006
Shape A	14 (7.3%)	3 (9.4%)		6 (7.2%)	0 (0%)	
Shape B	41 (21%)	5 (16%)		19 (23%)	0 (0%)	
Shape C	103 (53%)	12 (38%)		45 (54%)	4 (36%)	
Shape D	35 (18%)	12 (38%)		13 (16%)	7 (64%)	
Contact with the endplates, n (%)			0.054			0.035
Yes	7 (3.6%)	4 (12%)		1 (1.2%)	2 (18%)	
No	186 (96.4%)	28 (88%)		82 (98.8%)	9 (82%)	
Anti-osteoporotic treatment, n (%)			0.002			> 0.999
Yes	98 (51%)	7 (22%)		27 (33%)	4 (36%)	
No	95 (49%)	25 (78%)		56 (67%)	7 (64%)	
Scoliosis, n (%)			0.385			> 0.999
Yes	69 (36%)	14 (44%)		27 (33%)	4 (36%)	
No	124 (64%)	18 (56%)		56 (67%)	7 (64%)	
Preoperative AVH (mm)	19.5 ± 5.9	13.2 ± 5.4	< 0.001	19.3 ± 5.7	14.2 ± 3.9	0.001
Postoperative AVH (mm)	22.8 ± 5.3	18.6 ± 5.1	< 0.001	22.8 ± 4.8	19.4 ± 4.3	0.031
AVHRR, n(%)	1.43 ± 0.64	1.98 ± 0.63	< 0.001	1.45 ± 0.58	1.75 ± 0.43	0.055
Pre-op Cobb angle (°)	7 ± 7	9 ± 6	0.152	7 ± 5	8 ± 8	0.549
Post-op Cobb angle (°)	7 ± 7	8 ± 6	0.127	6 ± 5	8 ± 8	0.445
Cobb angle restoration (%)	0.31 ± 0.28	0.45 ± 0.24	0.004	0.34 ± 0.27	0.36 ± 0.18	0.728

*BMI* body mass index, *BMD* bone mineral density, *NVCFs* new vertebral compression fractures, *AVH* anterior vertebral height, *AVHRR* anterior vertebral height recovery ratio

### Construction of a Lasso-logistic regression-based prediction model

**Fig. 2:**
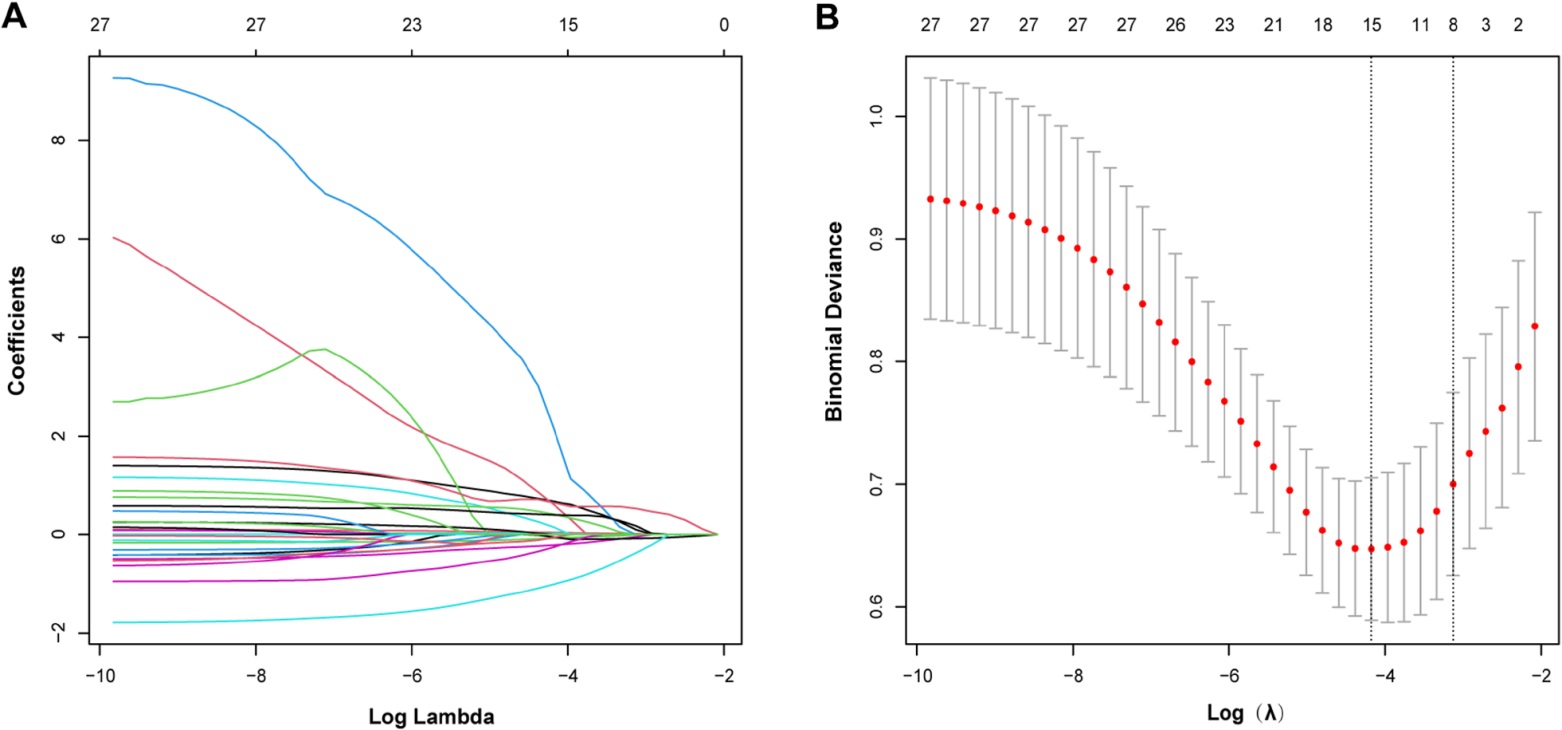
Filter variables based on lasso regression. **A** The variation characteristics of the variable coefficient. **B** The optimum value of λ in the lasso model is determined by ten-fold cross-validation

**Fig. 3:**
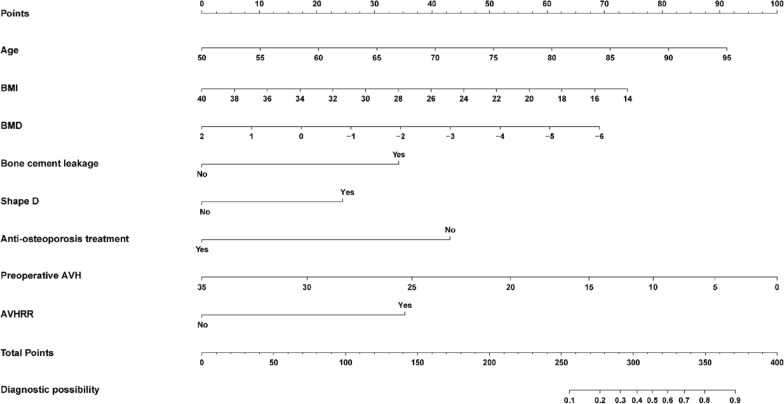
A nomogram is used to predict the risk of new vertebral compression fracture after PKP. BMI, body mass index; BMD, bone mineral density; PKP, percutaneous kyphoplasty; AVH, anterior vertebral height; AVHRR, anterior vertebral height recovery ratio

Variables were screened using Lasso regression. The changes in these variables and coefficients are shown in Fig. 2A. Iterative analysis was performed using the tenfold cross-validation method with λ = 0.043{Logλ =− 3.1} (Fig. 2) to obtain a model with good performance and the minimum number of variables. The screened variables included age, BMI, BMD, AVH, AVHRR, volume of bone cement leakage, anti-osteoporosis treatment status, and shape D (Fig. 1D, Table 2). A nomogram was created based on the screened variables (Fig. 3).

**Table 2:** Multivariate logistic regression analysis in the training cohort

	B	SE	Z	P	OR (95% CI)
Age	0.077	0.032	2.432	0.015	1.08 (1.015–1.15)
BMI	− 0.109	0.074	− 1.475	0.140	0.897 (0.776–1.037)
BMD	− 0.330	0.212	− 1.557	0.119	0.719 (0.475–1.09)
Bone cement leakage	1.305	0.523	2.495	0.013	3.688 (1.323–10.278)
Shape D	0.934	0.521	1.791	0.073	2.544 (0.916–7.062)
Anti-osteoporotic treatment	− 1.646	0.562	− 2.929	0.003	0.193 (0.064–0.58)
Preoperative AVH	− 0.036	0.133	− 0.270	0.787	0.965 (0.743–1.252)
AVHRR	1.347	0.716	1.882	0.060	3.845 (0.945–15.643)

*BMI* body mass index, *BMD* bone mineral density. *AVH* anterior vertebral height, *AVHRR* anterior vertebral height recovery ratio, *OR* odds ratio. *CI* confidence interval

### Model performance

**Fig. 4:**
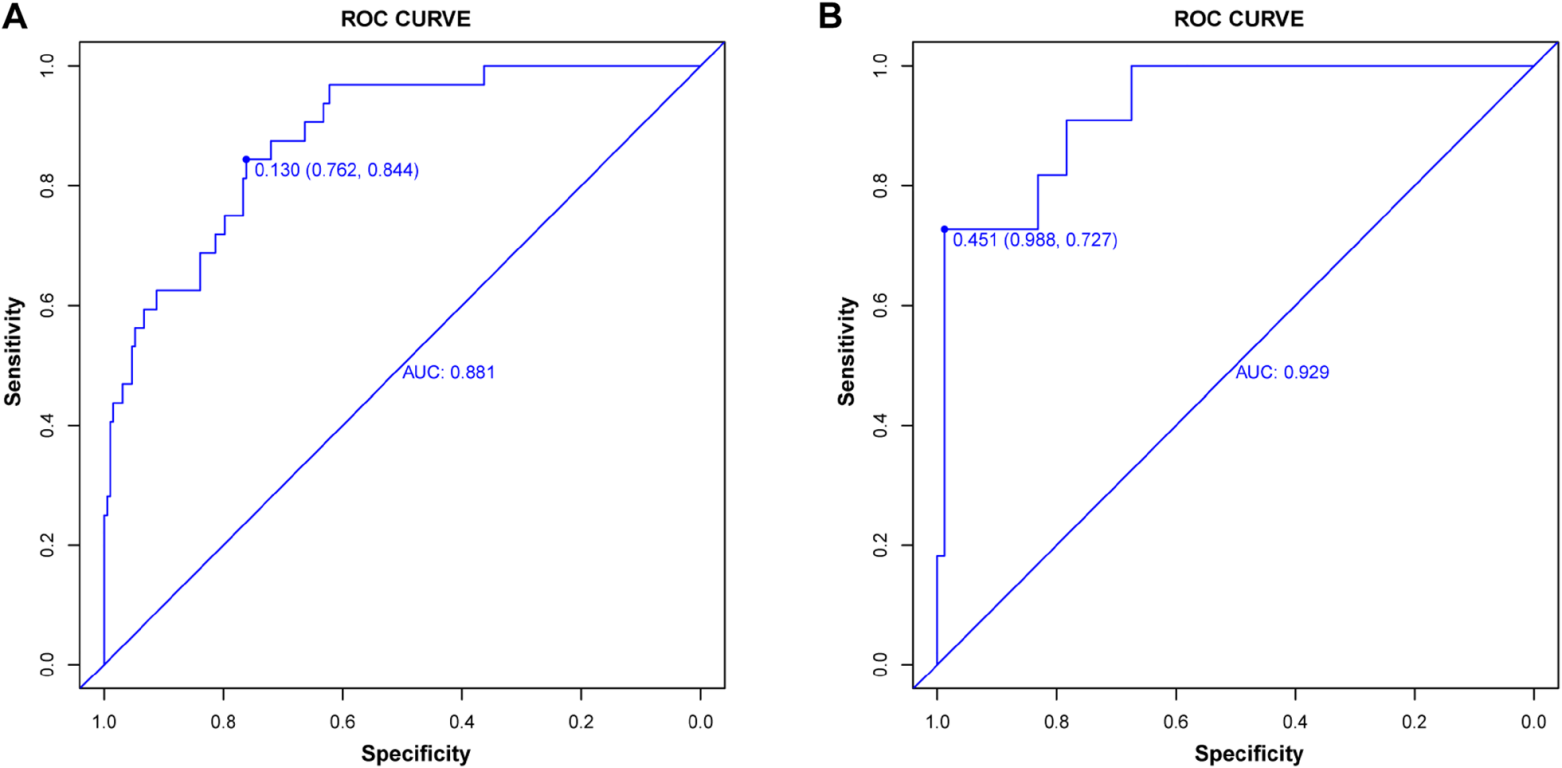
Receiver operating characteristic curves for the training **A** and validation **B** cohorts. *ROC* receiver operating characteristic, *AUC* area under the curve

**Fig. 5:**
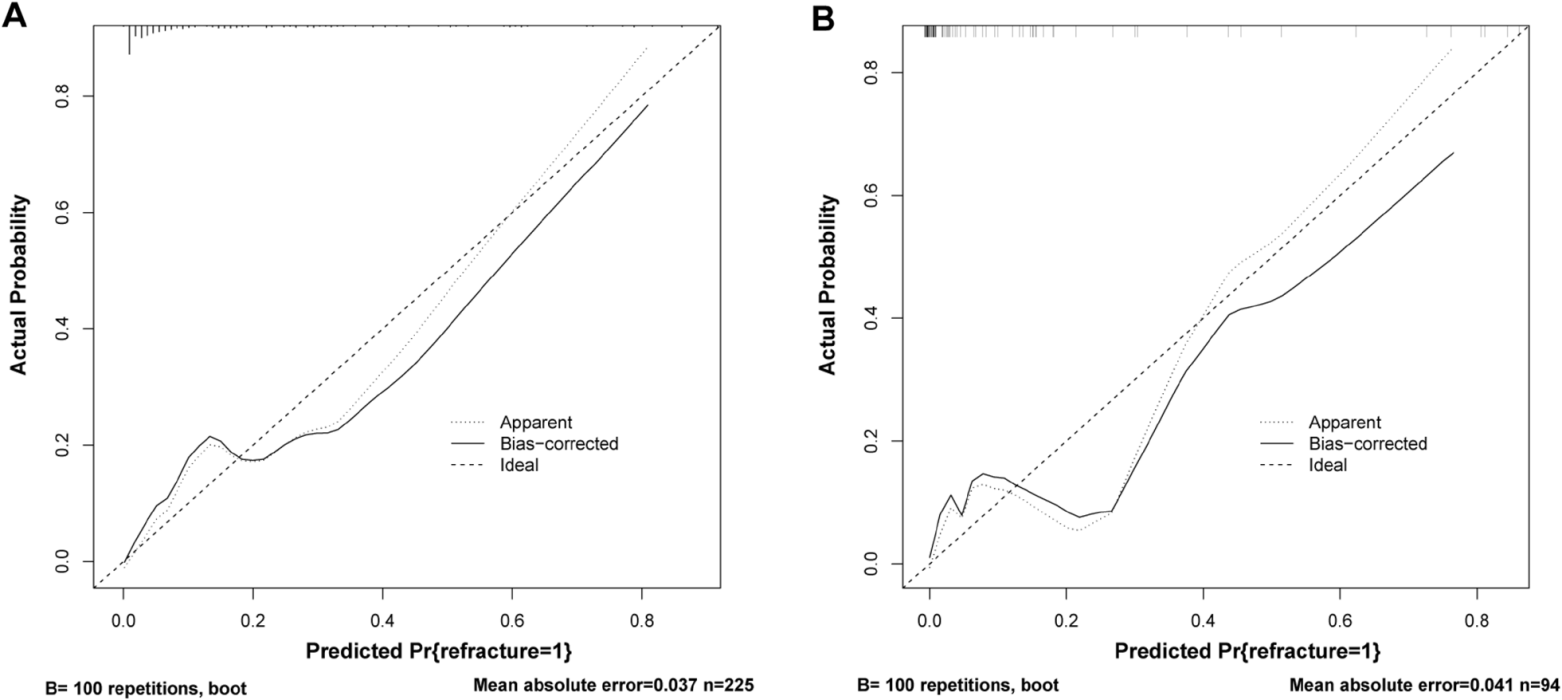
Calibration plots of predicted NVCFs based on logistic regression modeling in the training **A** and validation **B** cohorts. NVCFs, new vertebral compression fractures

**Fig. 6:**
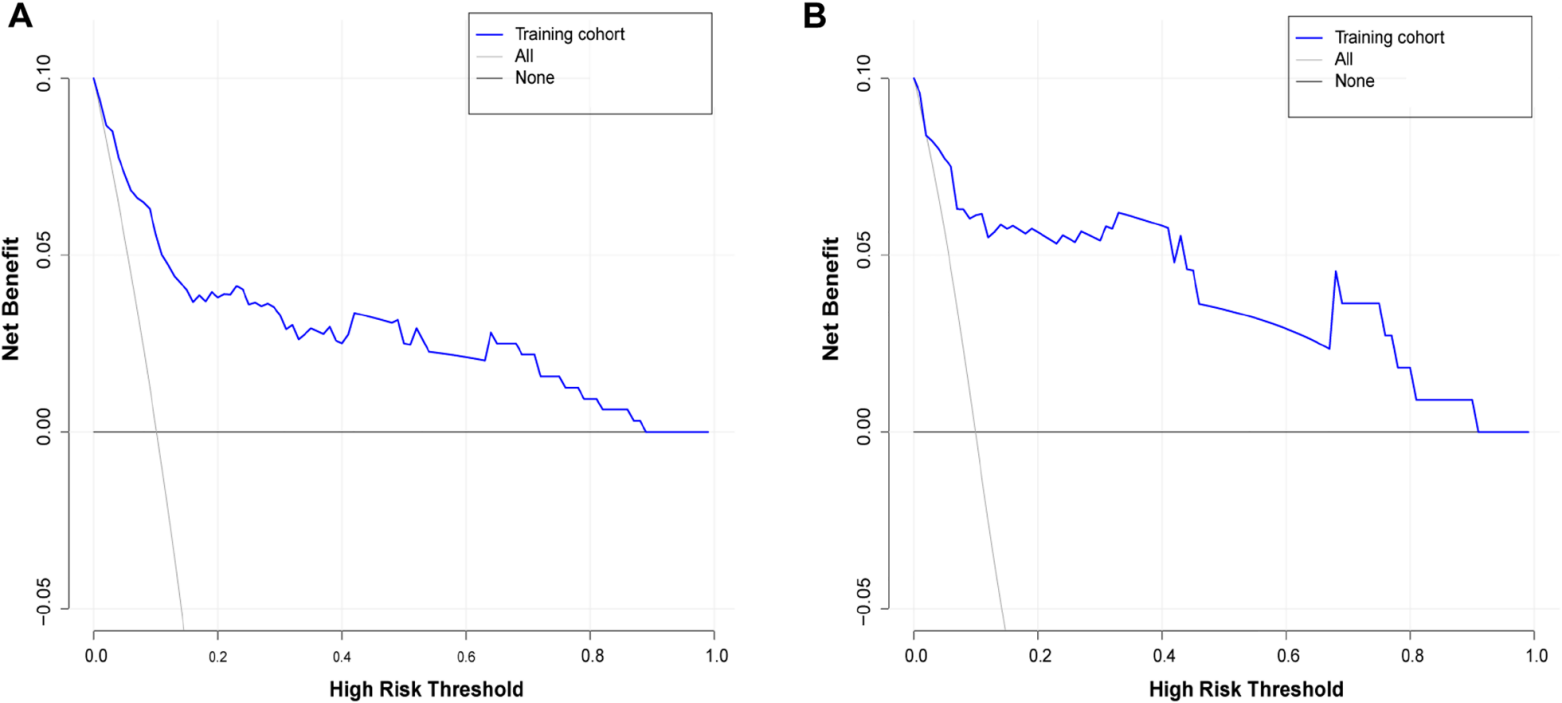
Decision curve analysis of nomogram prediction of NVCFs in the training **A** and validation **B** cohorts. NVCFs, new vertebral compression fractures

The receiver operating characteristic curve of the model was plotted (Fig. 4) to validate its discriminative ability. The area under the curve (AUC) of the training cohort was 0.881 (CI: 0.822–0.940), and the AUC of the validation cohort was 0.929 (CI: 0.857–1.000), indicating that the model has good discriminative ability. The calibration curves of the training and validation cohorts demonstrated that the predicted results of the model were in good agreement with the actual results (Fig. 5). In the training cohort, the decision curve analysis (DCA) curve showed that the model predicted the risk of NVCF with a net benefit at a threshold probability of 2–85% (Fig. 6A). In the validation cohort, the DCA curve showed that the model predicted the risk of NVCFs with a net benefit at a threshold probability of 1–88% (Fig. 6B). Overall, the model was feasible and appropriate for prediction.

## Discussion

OVCFs are common in patients with osteoporosis [13] and can be treated satisfactorily with minimally invasive surgery [14]. NVCFs are common and serious complications in patients with OVCFs that can lead to hospitalization. No studies have included BMD in prediction models; therefore, we developed and validated a nomogram in the present study to predict the risk of re-fracture after PKP surgery.

Osteoporosis is a systemic bone disorder characterized by low bone mass and destruction of bone tissue microarchitecture, leading to increased bone fragility and susceptibility to fracture [15]. The World Health Organization uses BMD and the T-score to define osteoporosis. The T-score is a standard deviation representing the difference between the patient and the mean BMD of healthy young adults. A T-score < − 2.5 is defined as osteoporosis, and a T-score between -1 and -2.5 is defined as osteopenia. The results of our multivariate analysis suggest that low BMD and age are independent risk factors for NVCF, and that BMD is negatively associated and age is positively associated with the development of NVCFs. In older women, increased bone remodeling in cancellous and cortical bone with negative remodeling balance leads to bone loss and destruction of bone microstructure. Cancellous bone exhibits trabecular thinning and trabecular loss, whereas cortical bone exhibits reduced cortical thickness and increased cortical porosity. In older men, osteoporosis is primarily associated with reduced bone formation and low bone turnover [16]. BMD decreases with age [17], and low BMD is a factor in surgical vertebral re-fracture that cannot be neglected [7]. An analysis of bone tissue and serum bone turnover markers in 206 patients by Qi et al*.* [18] showed that patients with low BMD had more necrotic bone tissue and lower bone turnover markers after fracture, indicating that patients with low BMD have poorer bone healing capacity. In addition, patients with osteoporosis have sparse trabeculae, significantly reduced vertebral body strength and compression resistance, and more severe vertebral body collapse under the same external force, making NVCFs more likely.

In our study, lack of anti-osteoporosis treatment is a major risk factor for development of NVCFs after PKP. Anti-osteoporosis treatment reduces the progression of osteoporosis and prevents the development of NVCFs [19]. In the training cohort, 98 control patients were given anti-osteoporosis treatment (51%), compared to seven NVCF patients (22%). Bisphosphonate and denosumab are first-line agents in the treatment of osteoporosis [20]. Bisphosphonate causes a progressive increase in BMD that plateaus after 3–4 years of treatment, whereas denosumab increases BMD more dramatically and persists for 10 years [20]. In a 3-year phase III clinical trial, denosumab reduced vertebral fractures by 68% [21]. Routine anti-osteoporosis treatment is recommended for post-PKP patients without contraindications.

Our study suggests that low BMI is a risk factor for NVCF. The effect of high BMI on vertebral fractures is controversial. In a study of osteoporotic fractures, high BMI was a protective factor for vertebral fracture due to the protective effect of higher adiposity [22]. Obese menopausal women tend to have higher estrogen levels, resulting in high BMD and low bone turnover and contributing to a lower risk of fracture [23]. However, it is hypothesized that obesity produces a pro-inflammatory/pro-oxidative state in bone, inhibiting bone formation and inducing bone resorption [24]. In addition, low BMI leads to NVCFs associated with low BMD [25]. Recent studies have suggested classifying Chinese adults with a BMI < 20 kg/m^2^ as malnourished [26]. Calcium and vitamin D are important nutritional factors in the management of osteoporosis. Calcium is an essential substance for bone mineralization and provides hardness and strength to bone [27]. Vitamin D regulates calcium homeostasis, and vitamin D deficiency also leads to osteomalacia. Studies have shown that malnutrition can promote the progression of osteoporosis [28].

In the present study, high AVHRR was considered an independent risk factor, consistent with previous findings [29–31]. In addition, we report for the first time that low AVH is also a risk factor. Patients with low preoperative vertebral body height tend to have higher vertebral body recovery rates. The relationship between high AVHRR and vertebral fracture has not been clearly explained. One hypothesis is that excessive vertebral body height recovery leads to increased tension of paravertebral soft tissues, which increases the mechanical load on the augmented vertebral body or the instability of the fractured segment [30]. Heo et al*.* [32] suggested that excessive vertebral body recovery may also increase the progression of osteonecrosis. PKP is not a procedure for correcting a deformity but rather a minimally invasive procedure used to reduce the pain experienced by patients with NVCFs. Therefore, moderate but not excessive expansion of the fractured vertebral body is recommended.

The simultaneous absence of contact between the bone cement and the upper and lower endplates, as well as the absence of cement leakage, were identified as independent risk factors for vertebral re-collapse. A retrospective study [33] found that NVCFs occurred 4.6 times more frequently in patients with bone cement leakage than in those without leakage. Other studies [34, 35] have confirmed that bone cement leaks through the ruptured endplates into the intervertebral disc, which results in altered peri-vertebral stresses and reduced disc cushioning. In addition, the distribution of bone cement in the treated vertebral body is considered a risk factor for vertebral re-fracture [36]. The results of a cohort study showed that adequate contact of bone cement with the upper and lower plates significantly reduced the risk of vertebral re-compression [37]. When bone cement contacted only the upper or the lower plate, the strength of the vertebral body was increased by only a factor of 2; however, when the cement contacted both the upper and lower plates, the strength was increased by a factor of 8–12, significantly improving stress transfer [38].

Lasso regression has the advantages of univariate analysis, as it can solve the problem of multicollinearity among variables. However, our study still has some limitations. First, the study was retrospective in nature and the effects of missing data and case selection bias were inevitable. Second, although the nomogram was validated in a validation cohort, the data were derived from the same hospital and were not validated through multiple centers in other regions and countries, which may limit the use of the model in some hospitals. Therefore, further validation in large-sample multi-center studies is needed in the future.

## Conclusions

In conclusion, the present study found that advanced age, low BMI, low BMD, lack of anti-osteoporosis treatment, low preoperative vertebral height, AVHRR ≥ 2, cement leakage, and shape D (lack of simultaneous contact of bone cement with the upper and lower plates) were independent risk factors for the development of NVCFs after PKP surgery. The nomogram developed in the present study is a good predictor of the risk of NVCFs after PKP. Physicians should develop individualized follow-up strategies based on risk, utilize clinical resources rationally, and avoid overtreatment.

## Abbreviations

NVCFs: New vertebral compression fractures
PKP: Percutaneous kyphoplasty
VCFs: Vertebral compression fractures
OCVFs: Osteoporotic vertebral compression fractures
BMD: Bone mineral density
BMI: Body mass index
AVH: Anterior vertebral height
AVHR: Anterior vertebral height ratio
AVHRR: Anterior vertebral height recovery ratio
AUC: The area under the curve
DCA: Decision curve analyses
